# Confirmation of a Gametophytic Self-Incompatibility in *Oryza longistaminata*

**DOI:** 10.3389/fpls.2021.576340

**Published:** 2021-03-31

**Authors:** Xiaoping Lian, Shilai Zhang, Guangfu Huang, Liyu Huang, Jing Zhang, Fengyi Hu

**Affiliations:** State Key Laboratory for Conservation and Utilization of Bio-Resources in Yunnan, Research Center for Perennial Rice Engineering and Technology of Yunnan, School of Agriculture, Yunnan University, Kunming, China

**Keywords:** gametophytic, *OlSP*, *OlSS*, *Oryza longistaminata*, self-incompatibility

## Abstract

*Oryza longistaminata*, a wild species of African origin, has been reported to exhibit self-incompatibility (SI). However, the genetic pattern of its SI remained unknown. In this study, we conducted self-pollination and reciprocal cross-pollination experiments to verify that *O. longistaminata* is a strictly self-incompatible species. The staining of pollen with aniline blue following self-pollination revealed that although pollen could germinate on the stigma, the pollen tube was unable to enter the style to complete pollination, thereby resulting in gametophytic self-incompatibility (GSI). *LpSDUF247*, a *S*-locus male determinant in the gametophytic SI system of perennial ryegrass, is predicted to encode a DUF247 protein. On the basic of chromosome alignment with *LpSDUF247*, we identified *OlSS1* and *OlSS2* as *Self-Incompatibility Stamen* candidate genes in *O. longistaminata*. Chromosome segment analysis revealed that the *Self-Incompatibility Pistil* candidate gene of *O. longistaminata* (*OlSP*) is a polymorphic gene located in a region flanking *OlSS1*. *OlSS1* was expressed mainly in the stamens, whereas *OlSS2* was expressed in both the stamens and pistils. *OlSP* was specifically highly expressed in the pistils, as revealed by RT-PCR and qRT-PCR analyses. Collectively, our observations indicate the occurrence of GSI in *O. longistaminata* and that this process is potentially controlled by *OlSS1*, *OlSS2*, and *OlSP*. These findings provide further insights into the genetic mechanisms underlying self-compatibility in plants.

## Introduction

Self-incompatibility (SI) is a reproductive mechanism of higher plants for preventing inbreeding by inhibiting self-pollination, and has been widely observed in Brassicaceae, Solanaceae, Rosaceae, Plantaginaceae, Scrophulariaceae, Gramineae, and Rutaceae (Pandey, 1960; Goring et al., 1993; Vaughan, 1994; Dickinson, 1995; McCormick, 1998; Liu et al., 2014; Iwano et al., 2015; Li et al., 2017; Ye et al., 2018; Liang et al., 2020). SI is described as a mechanism whereby plants are able to produce functional male and female gametes, but are incapable of producing seeds subsequent to self-pollination or the cross-pollination of plants containing SI alleles (Pandey, 1960; Takayama and Isogai, 2005). SI was divided into gametophytic self-incompatibility (GSI) and sporophytic self-incompatibility (SSI), which is defined based on the genetic mode of control of the incompatibility phenotype in pollen (de Nettancourt, 1997; McCubbin and Kao, 2000; Silva and Goring, 2001). In GSI, incompatibility is determined by pollen genotype, where growth inhibition of incompatible pollen occurs mainly in the style. In SSI, pollen grain incompatibility is determined by the diploid genome of the parent, in which mature pollen carry SI signal elements secreted by the tapetum, which lead to inhibiting of the germination of incompatible pollen on the stigma surface (de Nettancourt, 1997; Hiscock and Tabah, 2003; Hiscock and McInnis, 2004). The genetic mechanism of SI is dependent on signal transmission controlled by a single or multiple genetic loci between pollen and stigma (Franklin-Tong et al., 1988; Franklin-Tong and Franklin, 2003; Castric and Vekemans, 2004). The genetic loci associated with SI characteristically show high polymorphism, with respect to both the number of *S* alleles and differences in nucleotide sequences (Vekemans and Slatkin, 1994).

The SI system in Gramineae is GSI, which is thought to be controlled by two non-linked and polymorphic multi-allelic *S* and *Z* loci (Lundqvist, 1954; Cornish et al., 1979; Baumann et al., 2000; Thorogood et al., 2002). The *S* and *Z* loci in *Lolium perenne* L. have been found to be correspond to syntenic regions of chromosomes 5 and 4 in the genome of *O. sativa*, respectively (Yang et al., 2009). In total, 10 SI candidate genes have been identified in the SI cDNA libraries of *L. perenne*, and qRT-PCR analysis has revealed a rapid increase in the expression of these genes following pollen–stigma contact (Yang et al., 2009). Subsequently, nine candidate genes associated with SI were screened from *L. perenne* using molecular markers, and on the basis of expression profiling and nucleotide diversity assessment, two *Z*-linked genes, a *ubiquitin-specific protease* and a *LpDUF247* gene, have been identified as the most plausible candidates for the *S*-*Z* SI system (Shinozuka et al., 2010). In further studies, a total of 10,177 individuals of *L. perenne* from seven different mapping populations segregating for *S* locus showed a highly polymorphic gene encoding for a protein containing a DUF247 was fully predictive of known *S*-locus genotypes at the amino acid level (Manzanares et al., 2016). In addition, genome wide association studies and BLAST alignment with the *Brachypodium* physical map revealed highly significantly associated markers with peak associations from the chromosomal locations of candidate SI genes *S*- and *Z*-DUF247 (Thorogood et al., 2017). *LpSDUF247* is considered to be a *S*-locus male determinant in the gametophytic SI system that has five haplotypes with sequence polymorphism, which is a typical characteristic of SI. However, the molecular mechanisms underlying the regulation of SI in *L. perenne* have yet to be clarified (Thorogood et al., 2002; Shinozuka et al., 2010; Manzanares et al., 2016).

*Oryza longistaminata* is an AA-genome wild *Oryza* species of African origin that exhibits SI (Nayar, 1967). *O. longistaminata* has numerous desirable traits, such as long stigma and large anthers (Morishima, 1967), a rhizome (Ghesquiere, 2008), and high resistance to abiotic and biotic stresses (Khush et al., 1990; Song et al., 1995; Hu et al., 2003; Dong et al., 2017), and accordingly could represent a beneficial resource from the perspective of rice breeding. However, SI limits its free mating and hinders the exploitation of such desirable traits in rice cultivar improvement. In the present study, we identified a polymorphic *S*-locus associated with SI in this species, which will provide a basis for the determination of genetic patterns and molecular mechanisms of SI. Accordingly, we believe that the findings of this study could potentially facilitate exploitation of the desirable traits of *O. longistaminata*.

## Materials and Methods

### Plant Materials

For the determining the SI of *O. longistaminata*, we used the germplasms OlMK23 and OlMK68, which were originally collected from Ethiopia and were kindly provided by Dr. Melaku Getachew of the Institute of Biotechnology, College of Natural and Computational Sciences, Addis Ababa University, Ethiopia. For reciprocal hybrid testing, OlMK68 and OlMK23 plants were cultivated at Jinghong, Yunnan Province, China (21°59′N, 87 100°44′E, 611 m a.s.l.). For hybridization experiments, stamens were removed manually from the pollen recipients and mature pollen was collected from the pollen donors.

### Reciprocal Hybrids and Statistics

The *O. longistaminata* germplasms OlMK23 and OlMK68, and their reciprocal hybrids, each of which was represented by 10 samples, were cultivated for SI testing. For the reciprocal hybrids, the stamens were removed from a pollen recipient and mature pollen grains were collected from a pollen donor, after which plant were cross-pollinated and bagged. The seed-setting rate of OlMK23, OlMK68, and their reciprocal hybrids was determined after harvesting. Statistical analyses of seed-setting rate were based on the ratio between the number of harvested mature seeds and the total number of pollinated flowers in 10 independent pollination experiments.

### Aniline Blue Staining

At 5 min, 30 min, 1 h, and 2 h following self-/cross-pollination, more than 10 pollinated *O. longistaminata* and *O. sativa* pistils were collected and subsequently fixed overnight in Carnoy’s solution (90 mL of 50% ethanol, 5 mL of formaldehyde, and 5 mL of acetic acid). The pistils were then sequentially rehydrated with ethanol (50, 30, and 10%, each for 10 min) and then washed with deionized water. Thereafter, the pistils were softened in 1 M NaOH for 45 min at 55°C and stained with water-soluble aniline blue [0.1% (W/V) aniline blue and 0.1 M K_2_HPO_4_, pH 8.5] for 6 h in the dark at room temperature. The stained pistils were visualized by fluorescence microscopy.

### Identified and Syntenic Analysis of *OlSSs* and *OlSP*

*LpSDUF247* is a male component of the *S*-locus in *L. perenne* and is syntenic with *O. sativa* (Manzanares et al., 2016). *OsDUF247* (*Os05g0197900*), which corresponds to *LpSDUF247*, is located in the 6,030–6,090 kb region of *O. sativa* chromosome A05. Comparative genomics analyses were performed by comparing the genome sequences of *O. longistaminata* with chromosome 5 of *O. sativa*. *OlSS1* on Contig10 of *O. longistaminata* corresponds to *OsDUF247* (*Os05g0197900*). Sequence analysis was performed on the sequence flanking *OlSS1* to identify candidate polymorphic sequences. Haplotype and sequence polymorphism analyses of *OlSS1*, *OlSS2*, and *OlSP* were based on published transcriptome sequencing data (Zhang et al., 2015), whereas analysis of the conserved domain of OlSS1, OlSS2, and OlSP was performed using SMART^1^.

### RNA Isolation

The roots, stems, leaves, stamens, and pistils of *O. longistaminata* were used for tissue-specific expression analysis. The stamens were collected to determine the expression of *OlSS1* and *OlSS2* from stages 7 to 14 and expression in the pistils was determined from stages 9 to 14 as described by Zhang and Zoe (2009) for *O. sativa*. Total RNA was isolated from *O. longistaminata* using an Eastep^®^ Super Total RNA Extraction Kit (Promega, Shanghai, China), with first-strand cDNA being synthesized from 1 μg of the isolated RNA using a PrimeScript RT Reagent Kit with gDNA Eraser (TaKaRa BIO, Shiga, Japan).

### RT-PCR and qRT-PCR Analysis

The cDNAs of roots, stems, leaves, stamens, and pistils of *O. longistaminata* were used as templates for RT-PCR assays. RT-PCR and qRT-PCR amplifications were performed using the following primer pairs: *OlSS1*-1-F and *OlSS1*-1-R for *OlSS1*-1; *OlSS1*-2-F and *OlSS1*-2-R for *OlSS1*-2; *OlSS2*-1-F and *OlSS2*-1-R for *OlSS2*-1; *OlSS2*-2-F and *OlSS2*-2-R for *OlSS2*-2; *OlSP*-1-F and *OlSP*-1-R for *OlSP*-1; and *OlSP*-2-F and *OlSP*-2-R for *OlSP*-2. qRT-PCR analysis was performed using SYBR Premix Ex Taq Kit (TaKaRa BIO) in a Quant Studio 7 Flex real-time PCR system (Applied Biosystems, Foster City, CA, United States) with the following amplification program: initial denaturation at 95°C for 30 s, followed by 40 cycles of denaturation at 95°C for 5 s and primer annealing and extension at 58°C for 34 s. The results were normalized using the relative expression level of *Actin2* and analyzed using the 2^–Δ^ ^Δ^ ^*Ct*^ method (Livak and Schmittgen, 2001). Three biological replicates were performed for RT-PCR and qRT-PCR analysis. The sequences of the primers used in this study are presented in Supplementary Table 1.

### Homologous Cloning of *OlSSs* and *OlSP*

Relevant genomic and transcriptomic data for homologous cloning obtained from a previous study on *O. longistaminata* (Zhang et al., 2015). The cDNA sequences of *OlSSs* and *OlSP* were cloned from the transcriptomic database of *O. longistaminata* by homologous cloning (Zhang et al., 2015). For cDNA cloning, we used the following primers pairs: *OlSS1-1-cF* and *OlSS1-1-cR* were used for *OlSS1-1*; *OlSS1-2-cF* and *OlSS1-2-cR* for *OlSS1-2*; *OlSS2-1-cF* and *OlSS2-1-cR* for *OlSS2-1*; *OlSS2-2-cF* and *OlSS2-2-cR* for *OlSS2-2*; *OlSP-1-cF* and *OlSP-1-cR* for *OlSP-1*; and *OlSP-2-cF* and *OlSP-2-cR* for *OlSP-2*. The products thus amplified were inserted in the *pMD19-T* vector, and monoclonal sequencing was performed for different haplotypes.

## Results

### *Oryza longistaminata* Is a Strictly Self-Incompatible Species

The definitive characteristic of SI is that self-pollination of identical haplotypes results in sterility, whereas pollen is fertile when crossing different haplotypes (Franklin-Tong et al., 1988; Franklin-Tong and Franklin, 2003). In the present study, we grew plants of OlMK68 and OlMK23 in a field to evaluate the SI in *O. longistaminata* through self-pollination and reciprocal cross-pollination. Although the seed-setting rate of the hybrid (OlMK68/OlMK23) and its reciprocal hybrid was 20.5 ± 3.8 and 26.2 ± 4.7%, respectively (*n* = 10), in the case of both OlMK23 and OlMK68, self-pollination of plants failed to result in seed set, suggesting strict SI in this species (Table 1 and Supplementary Table 2).

**TABLE 1:** Seed-setting rate after self-/cross-pollination in *O. longistaminata*.

**Female**	**Male**	**Seed-setting rate (%)**
OlMK68	OlMK68	0
OlMK68	OlMK23	20.5 ± 3.8
OlMK23	OlMK23	0
OlMK23	OlMK68	26.2 ± 4.7

### *Oryza longistaminata* Exhibits Gametophytic Self-Incompatibility

**FIGURE 1:**
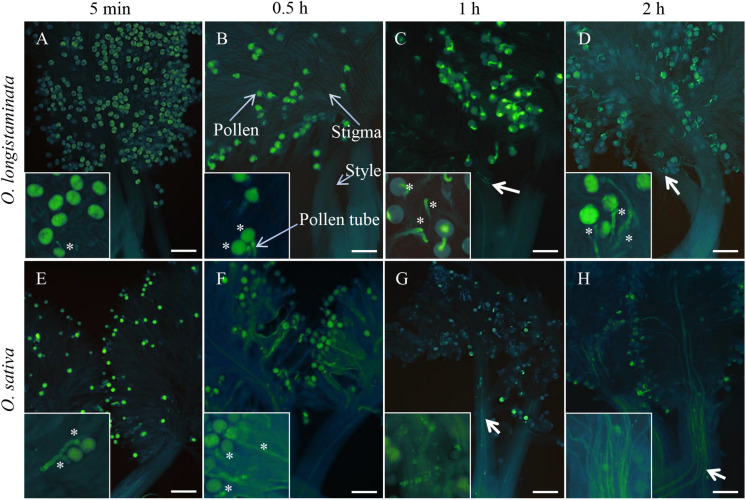
Pollen germination of *Oryza longistaminata* and *Oryza sativa*. **(A–D)** Self-pollination of *O. longistaminata*: **(A)** The pistils of self-pollinated OlMK68 after 5 min, stained with aniline blue solution. **(B)** The majority of pollen grains had germinated at 0.5 h following self-pollination. **(C)** The pollen tube reached the top of the style at 1 h after self-pollination. **(D)** At 2 h after pollination, the pollen tube remained on top of the style and appeared incapable of passing through the style. **(E–H)** Self-pollination of *O. sativa*: **(E)** Pollen began to germinate 5 min after self-pollination. **(F)** The pollen tube began to elongate at 0.5 h after self-pollination and passed through the style within 1–2 h following cross-pollination **(G,H)**. Bar = 50 μm. The asterisk indicates the germination of pollen grains. Bold arrows indicate pollen tubes in the style.

To determine pollen phenotype, we performed staining with aniline blue, which revealed that the pollen of *O. longistaminata* is able to germinate on the stigma after self-pollination (Figures 1A–D), with germination being observed within 5 min after self-pollination of OlMK68 (Figure 1A), a high rate of germination after 30 min, and the pollen tube reaching the top of the style within 1 h (Figures 1B,C). Subsequently, however, pollen tube growth was inhibited, with the pollen tube appearing to be incapable of passing through the style (Figure 1D). In contrast, pollen growth was consistent in self-pollinated of *O. sativa* after 5 and 30 min (Figures 1E,F), and the pollen tube was observed to have passed through the style within 1–2 h (Figures 1G,H). These results indicated that although the pollen of *O. longistaminata* is able to germinate on the stigma after self-pollination, it is incapable of subsequent passage through the style, there implying that this species exhibits GSI.

### *Oryza longistaminata* Self-Incompatibility Determinants Are Linked *S*-Loci

**FIGURE 2:**
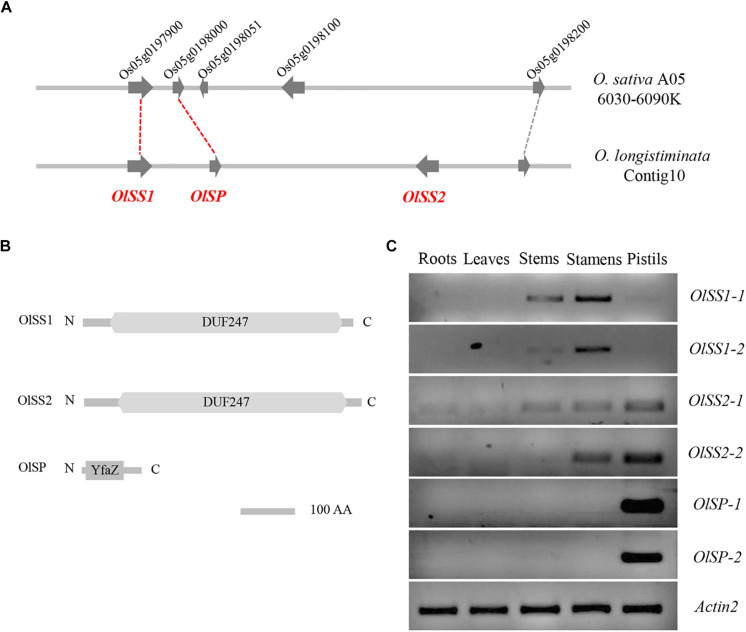
Syntenic and tissue expression of *OlSSs* and *OlSP*. **(A)** Syntenic representation of *OlSSs* and *OlSP* in *Oryza sativa* and *Oryza longistaminata*. *Os05g0197900*, which encodes an unknown DUF247 domain protein in *O. sativa*, corresponds to *OlSS1*, whereas *Os05g0198000* and *Os05g0198100* correspond to *OlSP* and *OlSS2* respectively. **(B)** Analysis of OlSS1, OlSS2, and OlSP protein domains. Both OlSS1 and OlSS2 contain a conserved DUF247 domain, whereas OlSP contains an N-terminal YfaZ domain of unknown function. **(C)** RT-PCR determination of the tissue-specific expression pattern of *OlSSs* and *OlSP* in *O. longistaminata*. *OlSS1-1* and *OlSS1-2* are mainly expressed in stamens, whereas *OlSS2-1* and *OlSS2-2* are expressed in both stamens and pistils, and *OlSP-1* and *OlSP-2* are highly and specifically expressed in pistils. *OlSS1*: *Self-incompatibility stamen1* from *O. longistaminata*; *OlSS2*: *Self-incompatibility stamen2* from *O. longistaminata*; *OlSP*: *Self-Incompatibility Pistil factor* from *O. longistaminata*; *O. sativa* A05: *Oryza sativa* chromosome 5.

The corresponding region of the *S*-locus of *L. perenne* was found on chromosome 5 of *O. sativa*, wherein a gene homolog of *LpSDUF247* is predicted to encode a DUF247 domain-containing protein (Manzanares et al., 2016). Comparative genomic analysis revealed that a gene located on contig10 of *O. longistaminata* is syntenic with *Os05g0197900* (*OsDUF247*), named *Self-Incompatibility Stamen1* of *O. longistaminata* (*OlSS1*) (Figure 2A). *OlSS1* encode a DUF247 domain protein, which is similar to LpSDUF247 and considered to be involved in SI (Figure 2B). On the basis of homologous sequence alignment, a gene was predicted to encode a protein containing a DUF247 domain about 19 kb downstream of *OlSS1*, named *Self-Incompatibility Stamen 2* of *O. longistaminata* (*OlSS2*) (Figures 2A,B). Previous genomic and transcriptomic sequencing has revealed that both *OlSS1* and *OlSS2* have two alleles in *O. longistaminata* (Zhang et al., 2015). RT-PCR assays indicated that alleles *OlSS1-1* and *OlSS1-2* are expressed primarily in the stamens, whereas the *OlSS2-1* and *OlSS2-2* alleles are expressed in both stamens and pistils (Figure 2C and Supplementary Figure 1). A defining characteristic of SI is that there should be a close linkage between male and female determinants and expression in stamens and pistils (Franklin-Tong, 2008). Both *OlSS1* and *OlSS2* show sequence polymorphisms and are expressed in stamens, we speculated that there is a *Self-Incompatibility Pistil factor* on contig10 of *O. longistaminata*. Indeed, subsequent sequence polymorphism analysis of a region flanking *OlSSs* revealed a candidate gene for such a factor, named *Self-Incompatibility Pistil factor* (*OlSP*), which may be involved in the SI of *O. longistaminata* (Figure 2A), given *OlSP* has a pair of alleles, *OlSP-1* and *OlSP-2*, that are highly and specifically expressed in the pistil (Figure 2C and Supplementary Figure 1). Structural analysis revealed that *OlSP* encodes an N-terminal YfaZ domain of unknown function (Figure 2B). Consequently, given that *OlSSs* and *OlSP* are closely linked and highly expressed in the stamens or pistils of *O. longistaminata*, we believe that *OlSSs* and *OlSP* could be candidate genes controlling SI in this species.

**FIGURE 3:**
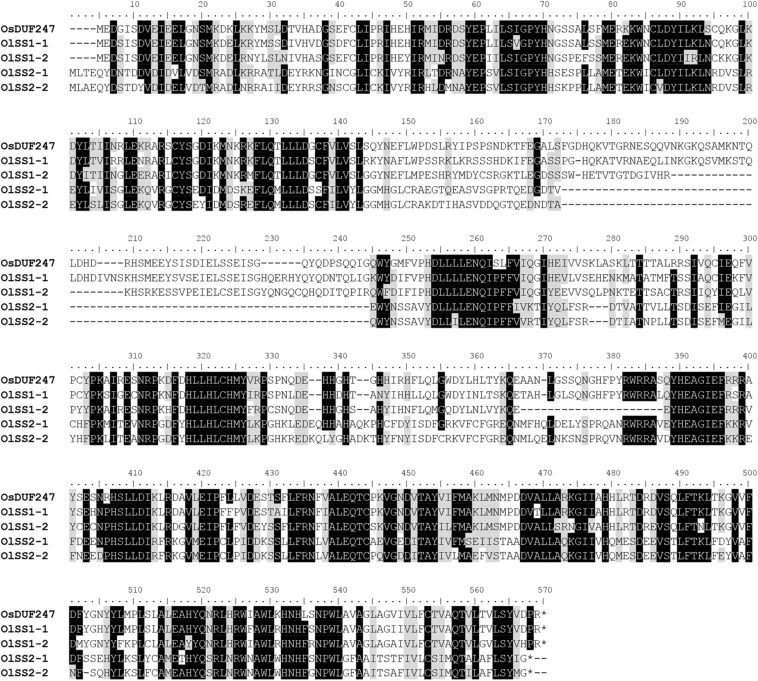
Amino acid sequence polymorphism of OlSSs. Alignment of the amino acid sequences of OlSS1-1 and OlSS1-2 with that of OsDUF247 revealed similarities of 80.7 and 69.8%, respectively, whereas the corresponding similarities of OlSS2-1 and OlSS2-2 are 38.9 and 36.9%, respectively. Both OlSS1 and OlSS2 showed conserved regions at the N and C termini.

**FIGURE 4:**
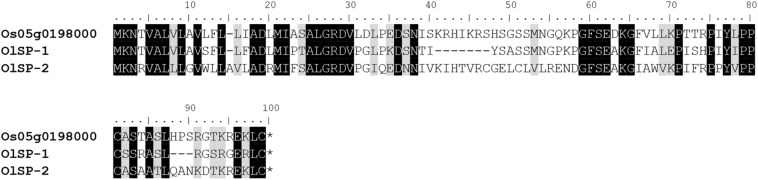
Amino acid sequence polymorphism of OlSP. OlSP-1 and OlSP-2 are the amino acid sequences encoded by the two alleles of *OlSP* in *Oryza longistaminata*. The sequence similarity between OlSP-1 and OlSP-2 and Os05g0198000 is 51.8 and 63.2%, respectively.

The alignment of OlSS1-1 and OlSS1-2 amino acid sequences with that of OsDUF247 exhibited similarity level of 80.7 and 69.8%, respectively (Figure 3), whereas the sequences of OlSS2-1 and OlSS2-2 showed lower similarities of 38.9 and 36.9%, respectively (Figure 3). Both OlSS1 and OlSS2 are characterized by conserved regions at the N and C termini. Furthermore, we established that similarities between the amino acid sequences of OlSP1 and OlSP2 and Os05g0198000 are 51.8 and 63.2%, respectively (Figure 4). These findings thus indicate that the *OlSS1*, *OlSS2*, and *OlSP* exhibit sequence polymorphism and can be considered putative SI candidate genes in *O. longistaminata*.

### *OlSSs* and *OlSP* Expression Patterns

**FIGURE 5:**
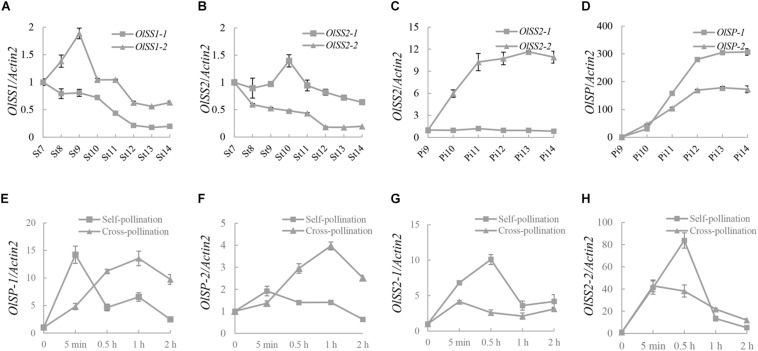
The expression pattern of *OlSSs* and *OlSP*. **(A)** Expression profile of *OlSS1* from stage 7 to stage 14 in stamens. *OlSS1-1* was down-regulated at all stamen developmental stages, whereas *OlSS1-2* was up-regulated at stages 7–9, and gradually down-regulated from stages 9 to 14. **(B)** The expression pattern of *OlSS2* from stage 7 to 14 in stamens. The down-related expression of *OlSS2-1* commenced at stage 10. **(C)** The expression pattern of *OlSS2* in pistils. *OlSS2-2* up-regulated during pistil development, whereas we detected no significant changes in the expression of *OlSS2-1*. **(D)** The expression profile of *OlSP* in pistils. *OlSP-1* and *OlSP-2* were up-regulated in pistils. **(E)** The expression profile of *OlSP-1* in pistils after self-/cross-pollination. The down-regulated expression of *OlSP-1* commenced 5 min after self-pollination, and reached the highest level at 1 h after cross-pollination. **(F)** The expression profile of *OlSP-2* in pistils after self-/cross-pollination. *OlSP-2* showed significant changes in expression following self-pollination, but was up-regulated after cross-pollination, attaining the highest level after 1 h. **(G)** The expression profile of *OlSS2-1* in pistils after self-/cross-pollination. *OlSS2-1* was up-regulated within 0.5 h of self-pollination, after which the levels of expression began to decline. In contrast, no significant changes were detected in the expression of *OlSS2-1* following cross-pollination. **(H)** The expression profile of *OlSS2-2* in pistils after self-/cross-pollination. *OlSS2-2* was up-regulated within 0.5 h of self-pollination, and subsequently down-regulated. Following cross-pollination, the highest level of *OlSS2-2* expression was detected within 5 min, after which expression was down-regulated. St, stamen; Pi, pistil; St7 to St14, pollen development from stages 7 to 14; Pi9 to Pi14, pistil development from stages 9 to 14.

The expression levels of *OlSS1*, *OlSS2*, and *OlSP* at different development stages of stamen and pistil were analyzed by qRT-PCR assay. The expression of *OlSS1-1* was down-regulated during all developmental stages of the stamens, whereas the expression of *OlSS1-2* was up-regulated in stages 7–9 and thereafter gradually down-regulated in stages 9–14 (Figure 5A). Interestingly, the expression *OlSS2* in stamens was found to show a pattern similar to that of *OlSS1*, with the down-regulated expression of *OlSS2-1* started commencing from stage 10 (Figure 5B). Of the two *OlSS2* alleles, whereas *OlSS2-2* was up-regulated during pistil development but the expression of *OlSS2-1* no significant change (Figure 5C). Moreover, both *OlSP-1* and *OlSP-2* were rapidly up-regulated during pistil development, with highest expression levels being detected at stage 13 (Figure 5D).

Analysis of the expression profiles of *OlSP-1*, *OlSP-2*, *OlSS2-1*, and *OlSS2-2* after self-/cross-pollination revealed that the expression of *OlSP-1* was highest 5 min after pollination and increased gradually within 1 h after cross-pollination (Figure 5E). Contrastingly, *OlSP-2* exhibited no significant change following self-pollination, although showed an expression pattern similar to that of *OlSP-1* following cross-pollination (Figure 5F). Expression of *OlSS2-1* was found to be highest at 30 min after self-pollination, whereas no significant change following cross-pollination (Figure 5G). *OlSS2-2* was up-regulated within 30 min and subsequently down-regulated after self-pollination, whereas following cross-pollination, highest expression levels were detected within 5 min, after which expression was down-regulated (Figure 5H). These observations indicate that whereas *OlSS1* and *OlSP* are expressed in the stamens and pistils, respectively, *OlSS2* is expressed in both stamens and pistils, thereby indicating that these three genes are associated with stamen and pistil development. In addition, the results revealed that *OlSS1*, *OlSS2*, and *OlSP* have different expression patterns after self-/cross-pollination.

## Discussion

Studies on the genetics of SI in Gramineae were mainly conducted on *Secale cereale* and *Phalaris coerulescens*, and have indicated that SI in grass species is under gametophytic control mediated by two unlinked multi-allelic loci referred to as *S* and *Z* (Lundqvist, 1954, 1956). Available data indicate that the two-locus SI system is common to all self-incompatible grass species of the subfamily Pooideae, and possibly to all members of the family Gramineae (Connor, 1979). The most significant differences with regards to SI are reciprocal differences in compatibility between two parents (Leach, 1988). In the present study, examination of the compatibility between the two investigated *O. longistaminata* germplasms (OlMK68 and OlMK23) revealed complete infertility following self-pollination and different seed-setting rates in reciprocal hybrids, indicating strict SI.

In SI, the transmission of signals between the stamens and pistils is dependent on the interaction of *S*-haplotypes with polymorphic sequences (Franklin-Tong et al., 1988; Franklin-Tong and Franklin, 2003). Therefore, putative specificity determinants should meet the following three criteria suggested by the biology and genetics of SI: linkage to the *S*-locus, polymorphism between different *S*-haplotypes, and expression in the pollen or pistil (Franklin-Tong, 2008). In the present study, the sequence polymorphisms and expression patterns of *OlSSs* and *OlSP* were in accordance with the characteristics of SI. Moreover, *OlSSs* and *OlSP* were found to be closely linked on the *O. longistaminata* genome, which is consistent with the signaling mechanism of SI.

The primer pairs used in this study were designed based on the allele sequences of *OlSSs* and *OlSP* from OlMK68, and on the basis of the tissue-specific expression and expression levels in stamens and pistils at different stages of developmental, we confirmed that *OlSSs* and *OlSP* are polymorphic loci. Moreover, qRT-PCR analysis revealed that the alleles of *OlSS1* and *OlSS2* show identical expression patterns in stamens, thereby indicating the comparable or synergistic functions of *OlSS1* and *OlSS2*. Given that the expression of *OlSS1* was gradually down-regulated during the latter stages of pollen maturation, further examination of the expression of this gene at the cellular level will be necessary to more precisely determine whether *OlSS1* is expressed in the pollen or in the tapetum. Besides, *OlSS2* was also expressed in the pistil, but only the *OlSS2-2* allele is up-regulated during pistil development. The reason for this phenomenon could be indictive of the complex mechanism of SI in *O. longistaminata*, which requires further study. A notable observation in the present study was that pollen on the stigma was unable to enter the style following self-pollination, indicating that either the pollen carries a male SI signal factor and/or that a pistil signal factor is secreted into the style. Although *OlSSs* and *OlSP* are considered to be the respective candidate genes for these factors in the SI of *O. longistaminata*, the mechanisms whereby they contribute to the regulation SI need to be further studied.

## Data Availability Statement

The raw data supporting the conclusions of this article will be made available by the authors, without undue reservation.

## Author Contributions

FH and XL designed the experiments and wrote the manuscript. XL, SZ, and JZ performed the experiments. GH and LH analyzed the data. All authors have read and agreed to the submitted version of the manuscript.

## Conflict of Interest

The authors declare that the research was conducted in the absence of any commercial or financial relationships that could be construed as a potential conflict of interest.

## Footnotes

1 http://smart.embl.de/
