# Supplementary material for: Confirmation of a Gametophytic Self-Incompatibility in Oryza longistaminata
Source: Front Plant Sci. 2021 Mar 31;12:576340. doi: 10.3389/fpls.2021.576340 (PMC8044821; doi:10.3389/fpls.2021.576340)
Supplement: Supplementary file 1 [file Data_Sheet_1.docx]

**Supplementary Table S1: List of Primers used for this manuscript**

| Name | Sequence (5'-3') | Name | Sequence (5'-3') |
| --- | --- | --- | --- |
| *OlSS1-1-F* | CTAGTTCCCGCAAACTTAAGCG | *OlSS1-1-cF* | GCTCTGTCGATCAGTTAAGAGG |
| *OlSS1-1-R* | CTTTCCAATCAGCTGAGTGTTG | *OlSS1-1-cR* | CCTTGGATCAACATAAGACAAGACAGT |
| *OlSS1-2-F* | AACTAGTGCGTGCACAAGAAGC | *OlSS1-2-cF* | GCATGTAACTAGTGTAAAGGTTTTAAG |
| *OlSS1-2-R* | GAAAGCTGGAATATTCATCC | *OlSS1-2-cR* | CCTGGGATGAACATAAGATAAGACACC |
| *OlSS2-1-F* | GACGGTGACACTGTAGAATG | *OlSS2-1-cF* | ATGCTGACTGAACAATACGAC |
| *OlSS2-1-R* | CAGTAGTTGCTACCGTATCACGA | *OlSS2-1-cR* | TATGTATGATAAGAAAGCAAGAGC |
| *OlSS2-2-F* | TGATACCATAGCAACTAATCCA | *OlSS2-2-cF* | ATGCTGGCCGAACAATATGACAG |
| *OlSS2-2-R* | ATAAAGTTGTTTATCCTCCCG | *OlSS2-2-cR* | CATGTACGATAAGAAAGCAAGAAT |
| *OlSP-1-F* | TAGCGGTTTTGGCTGATCG | *OlSP-1-cF* | ATGAAGAACACGGTCGCTCTCG |
| *OlSP-1-R* | TGTCCTTGTTAGCCTGTAAG | *OlSP-1-cR* | TTAACACAGCCTTTCTCCCCTG |
| *OlSP-2-F* | TGATGATCCCTACTGCTCTTG | *OlSP-2-cF* | ATGAAGAACAGAGTCGCCCTGC |
| *OlSP-2-R* | ATAGGATGGGAAATCGGTTC | *OlSP-2-cR* | GCGAAGGGCATCGCTTGGGTGA |

**The original data about Table 1**

**Supplementary Table 2 |** Setting rate after self-/ cross-pollination in *O. Longistaminata*

| Number | Female parent | Male parent | Seed setting rate (%) |
| --- | --- | --- | --- |
| A-1 | OlMK68 | OlMK68 | 0 |
| A-2 | OlMK68 | OlMK68 | 0 |
| A-3 | OlMK68 | OlMK68 | 0 |
| A-4 | OlMK68 | OlMK68 | 0 |
| A-5 | OlMK68 | OlMK68 | 0 |
| A-6 | OlMK68 | OlMK68 | 0 |
| A-7 | OlMK68 | OlMK68 | 0 |
| A-8 | OlMK68 | OlMK68 | 0 |
| A-9 | OlMK68 | OlMK68 | 0 |
| A-10 | OlMK68 | OlMK68 | 0 |
| B-1 | OlMK68 | OlMK23 | 21.9 |
| B-2 | OlMK68 | OlMK23 | 16.7 |
| B-3 | OlMK68 | OlMK23 | 27.6 |
| B-4 | OlMK68 | OlMK23 | 18.4 |
| B-5 | OlMK68 | OlMK23 | 26.4 |
| B-6 | OlMK68 | OlMK23 | 18.1 |
| B-7 | OlMK68 | OlMK23 | 17.9 |
| B-8 | OlMK68 | OlMK23 | 20.5 |
| B-9 | OlMK68 | OlMK23 | 16.9 |
| B-10 | OlMK68 | OlMK23 | 21.0 |
| C-1 | OlMK23 | OlMK23 | 0 |
| C-2 | OlMK23 | OlMK23 | 0 |
| C-3 | OlMK23 | OlMK23 | 0 |
| C-4 | OlMK23 | OlMK23 | 0 |
| C-5 | OlMK23 | OlMK23 | 0 |
| C-6 | OlMK23 | OlMK23 | 0 |
| C-7 | OlMK23 | OlMK23 | 0 |
| C-8 | OlMK23 | OlMK23 | 0 |
| C-9 | OlMK23 | OlMK23 | 0 |
| C-10 | OlMK23 | OlMK23 | 0 |
| D-1 | OlMK23 | OlMK68 | 30.6 |
| D-2 | OlMK23 | OlMK68 | 28.4 |
| D-3 | OlMK23 | OlMK68 | 24.4 |
| D-4 | OlMK23 | OlMK68 | 19.3 |
| D-5 | OlMK23 | OlMK68 | 27.1 |
| D-6 | OlMK23 | OlMK68 | 23.4 |
| D-7 | OlMK23 | OlMK68 | 29.5 |
| D-8 | OlMK23 | OlMK68 | 33.0 |
| D-9 | OlMK23 | OlMK68 | 27.7 |
| D-10 | OlMK23 | OlMK68 | 18.5 |

**
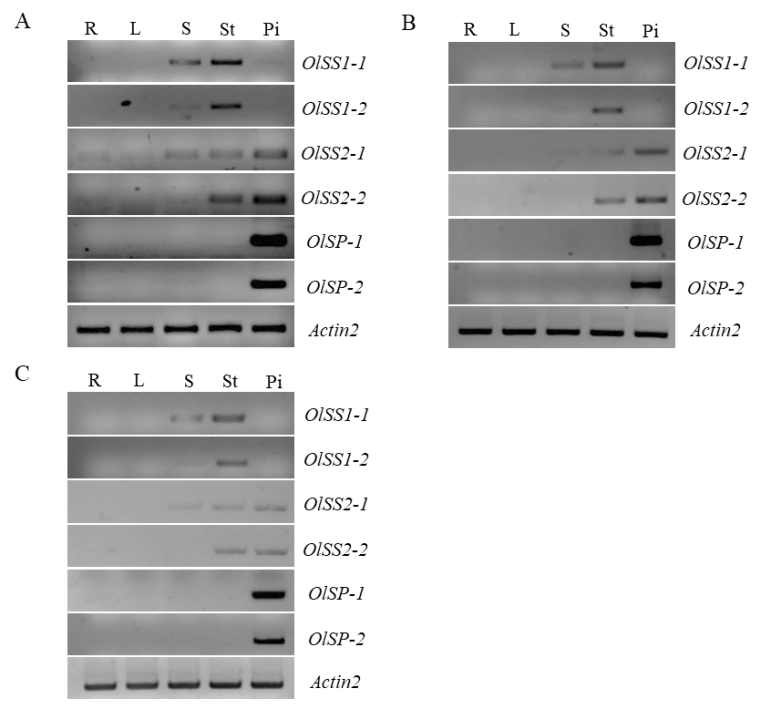
The original data about Figure 2C**

**Supplemental Figure 1 |** Tissue expression of *OlSSs* and *OlSP*. Three biological replicates were performed for the tissue expression. (**A**), (**B**) and (**C**) represent three independent replicates of RT-PCT. R: roots, L: leaves, S: stems, St: stamens, Pi: pistils, *OlSS1*: *Self-incompatibility stamen1* from *O. longistaminata*; *OlSS2*: *Self-incompatibility stamen2* from *O. longistaminata*; *OlSP*: *Self-Incompatibility Pistil factor* from *O. longistaminata*.
